# Initial Smoking Experiences and Current Smoking Behaviors and Perceptions among Current Smokers

**DOI:** 10.1155/2013/491797

**Published:** 2013-09-12

**Authors:** Hugh Klein, Claire E. Sterk, Kirk W. Elifson

**Affiliations:** ^1^Kensington Research Institute, 401 Schuyler Road, Silver Spring, Maryland, MD 20910, USA; ^2^Rollins School of Public Health, Emory University, Atlanta, GA 30322, USA

## Abstract

*Purpose*. We examine early-onset cigarette smoking and how, if at all, it is related to subsequent smoking practices. *Methods*. From 2004 to 2007, face-to-face interviews were conducted with 485 adult cigarette smokers residing in the Atlanta metropolitan area. Data analysis involved a multivariate analysis to determine whether age of smoking onset was related to current smoking practices when the effects of gender, age, race, marital/relationship status, income, and educational attainment were taken into account. *Results*. The mean age for smoking onset was 14.8, and more than one-half of all smokers had their first cigarette between the ages of 12 and 16. Most people reported an interval of less than one month between their first and second time using tobacco. Earlier onset cigarette smoking was related to more cigarette use and worse tobacco-related health outcomes in adulthood. *Conclusions*. Early prevention and intervention are needed to avoid early-onset smoking behaviors. Intervening after initial experimentation but before patterned smoking practices are established will be challenging, as the interval between initial and subsequent use tends to be short.

## 1. Introduction

 Research focusing on the age of initiation for various types of legal and illegal substances has shown that “average” Americans begin experimenting with substances, typically alcohol and/or tobacco, during their teenage years [1]. Increasingly, early-onset use appears to be occurring. By the time they are nine or ten years old, approximately 10% of the American children have begun drinking alcohol [2] and nearly one-third of all youths begin drinking prior to the age of thirteen [3]. By 10th grade (approximately aged 15 or 16), more than one-half (58.2%) of all American youths have used alcohol [1]; and by 12th grade (approximately aged 17 or 18), more than one-half (54.1%) of American adolescents have been drunk at least once [1]. Study findings have revealed that early-onset alcohol use oftentimes is associated with a greater likelihood of subsequent illegal drug use [4]. 

 When it occurs, experimentation with illegal drugs typically begins, on average, a few years after initial alcohol consumption. Recent data suggest that 28.6% of 13 and 14 year olds, 40.6% of 15 and 16 year olds, and 49.9% of 17 and 18 year olds have used at least one illegal drug during their lifetime [1]. By far, the most commonly used of these drugs is marijuana. The median age of first marijuana use is 15.5 years [5]. Nearly two-thirds (62.6%) of all marijuana users first try the substance between the ages of 13 and 17, and the large majority of these persons (90.9%) report prior alcohol and/or tobacco use [5]. Early onset of marijuana use (defined here as experimentation prior to age 15) has been shown to be related to daily marijuana use as well as the use of heroin, cocaine, and other illegal drugs in adulthood [5, 6].

 It is not uncommon for American youths to experiment with other types of drug use. While experimentation with cigarette smoking has declined sharply among young people since the 1990s [1], it remains the third ranked substance of experimentation among American adolescents, ranking only behind alcohol and marijuana. The average age of smoking initiation in the United States has been reported to be approximately 17 or 18 years [7]. In 2010, nearly one-half (42.2%) of all 12th graders reported having smoked a cigarette, with one-sixth (17.6%) reporting having tried some type of smokeless tobacco during their lifetime [1]. Approximately one-half of those who ever smoked reported having done so during the past month, and among them, approximately one-half smoked daily [1]. When all types of tobacco products are combined, more than one-quarter (26.0%) of all high school students are current users of tobacco [8]. 

 Research on how age of initiation of cigarette smoking relates to subsequent smoking behaviors is sparse. In one study of early-onset cigarette smokers (defined as persons initiating use prior to age 12), about three-quarters of the young people (77.0%) became regular smokers by mid-adolescence [9]. Other researchers found that compared to people who began smoking at a later age, those who began smoking prior to age 16 were less than one-half as likely to quit smoking [10]. In their research on smoking initiation during the college years, Clarkin and colleagues [11] found that about one out of nine (11.7%) students had their first cigarette while in college years and 10.8% of college students began smoking regularly at some point during their college years. Having a positive experience (e.g., experiencing relaxation) when first smoking a cigarette has been associated with an increased risk of current smoking, daily smoking, nicotine dependence, and cue-induced cravings for a cigarette [12]. 

 In this paper, using a community-based sample of adult current cigarette smokers, we examine (1) the age of onset of cigarette smoking, (2) people's recollections of their first smoking experiences, and (3) the potential link between age of onset and subsequent cigarette smoking behaviors. In doing so, we aim to add to the literature on smoking initiation and address important lessons that can be learned for prevention and intervention efforts.

## 2. Methods

### 2.1. Subjects and Design

The data presented in this paper are part of the Persistent Smokers Project (PSP) in Atlanta, Georgia. The community-based sample of 485 current smokers is distinct from those reported in many other studies, which have been based on younger, school-based research populations or those recruited at clinics or other institutional settings. Data were collected between September 2004 and July 2007. To be eligible, the participants had to be aged 18 years or older and reside in the Atlanta metropolitan area. Study participants had to have smoked at least 100 cigarettes during their lifetime (which is consistent with the National Health Interview Survey (NHIS) classification [13] as “ever smoked”) and have smoked in the last week (which is consistent with the NHIS classification [13] as “current smoker”). 

 Participant recruitment involved purposive sampling, including a combination of active and passive recruitment techniques. Using a short screening form, potential study participants were screened in the setting where they were recruited, such as near office buildings or other work locations, at restaurants, in social entertainment settings, in parks, and in other public settings. Passive recruitment involved posting flyers in local venues such as stores, restaurants, and community centers. Interested individuals, who called the project phone line listed on the flyers, initially were screened over the phone using the same short form used in the active recruitment. Two-thirds of the respondents (*n* = 325) were brought into the study via active recruitment, with the remaining one-third being identified through passive recruitment (*n* = 160). We did not identify any significant difference in the sample characteristics or in the outcome variables based on the recruitment strategy. 

 Once a person was identified as eligible, the staff member described the study and time required to participate. The most common reason for ineligibility was having smoked an insufficient number of cigarettes to qualify, either during the person's lifetime or during the preceding week. The interviews took place at a mutually convenient location, such as one of the project offices, the respondent's home, a local restaurant or coffee shop, or community centers. Additional information was provided on the nature of the study, the time required, and the informed consent and other confidentiality procedures. The questionnaire contained items covering the respondent's social background characteristics, smoking behaviors, attitudes, and opinions, as well as items about alcohol and other drugs, a health inventory, and self and identity items. The average length of the interview was 90 minutes and respondents received $20 as compensation for their time and participation. Prior to implementation in the field, all study protocols were reviewed and approved by the institutional review board at Emory University and Georgia State University.

### 2.2. Measures Used

Three variables focused on initial smoking behaviors: Age of onset of cigarette smoking was a continuous measure assessed by respondents' answers to the question “How old were you when you smoked your first cigarette?” This question was asked separately from one in which respondents were asked when they had their first puff or two from a cigarette. Age of first cigarette purchase was measured similarly. The amount of time that elapsed between the first and the second time a person smoked was a seven-level ordinal measure, with the following response options: (1) “less than a day,” (2) “one day,” (3) “more than one day but less than a week,” (4) “one week but less than one month,” (5) “one month but less than three months,” (6) “three months but less than twelve months,” and (7) “one year or more.” All of these measures (and all other measures of earlier-life smoking behaviors) were based on retrospective reports which, by their very nature, cannot be corroborated.

Initial smoking experiences were assessed using the following measures: source of first cigarette was a categorical measure asking respondents to indicate how they obtained their first cigarette: bought a cigarette on their own, got a cigarette from another person (e.g., parent, sibling, other relative, or friend), took/stole a cigarette from someone (e.g., parent, sibling, other relative, or friend), or some other way. Reality versus expectations regarding the first smoking experience was a categorical measure in which study participants selected one of the following response choices: more negative than expected, just as expected, or more positive than expected. Recollection of the first smoking experience included yes/no responses to items identified in our formative research on smoking initiation: coughing, feeling calm or relaxed, feeling dizzy, having more energy, feeling panicked, and so forth. Reasons for smoking again after the initial cigarette included yes/no responses to items such as: (a) I liked what it did for me, (b) I liked how it tasted, (c) I thought the next time would be better than the first time, (d) a friend offered it to me and I felt I could not decline, (e) it was cool to smoke, and (f) I wanted to be with friends, all of whom were smokers. 

Current smoking behaviors were assessed with several measures. Number of cigarettes smoked was assessed based on the number of cigarettes smoked per week (continuous measure). This was computed from the number of days respondents reported having smoked during the preceding month, the average number of cigarettes smoked on a typical weekday, and the average number of cigarettes smoked over the course of a typical weekend. Smoking while ill was assessed from a question that asked “Do you smoke if you are so ill that you are in bed most of the day?” The response choices were “no” (coded as 0), “much less” (coded as 1), “somewhat less” (coded as 2), “the same as when I am not ill and need to stay in bed” (coded as 3), “a little more” (coded as 4), and “much more” (coded as 5). Needing a cigarette to function was derived from the question “How often do you feel that you need a cigarette to help you function?” Response choices were: “never” (coded as 0), “less than once a month” (coded as 1), “about once a month” (coded as 2), “a few times a month” (coded as 3), “about once a week” (coded as 4), “several times a week” (coded as 5), “daily” (coded as 6), and “every 2-3 hours or more often” (coded as 7). Taking a special trip to get cigarettes involved the question “How often do you make special trips to get cigarettes?” Chain smoking frequency was assessed by asking “How often do you chain-smoke, that is, smoke one cigarette right after another?” Smoking more than intended was derived from the question “How often do you smoke more cigarettes than you intend to smoke?” Response choices for these last three measures were “never” (coded as 0), “less than once a month” (coded as 1), “about once a month” (coded as 2), “a few times a month” (coded as 3), “about once a week” (coded as 4), “several times a week” (coded as 5), and “daily” (coded as 6). 

Perceptions related to smoking were examined with several measures. Perceived benefits of smokingwere measured using a scale consisting of twelve items that were adapted from the work of Myers and colleagues [14] and Copeland and colleagues [15]. All items were scored using a five-point Likert scale, with responses ranging from “strongly disagree” to “strongly agree.” Items comprising the perceived benefits scale included: (a) “When I'm angry, smoking a cigarette calms me down”; (b) “Smoking calms me down when I feel nervous”; (c) “Smoking energizes me”; (d) “Cigarettes can really make me feel good”; (e) “When I am worrying about something, smoking a cigarette is helpful”; (f) “When I'm feeling happy, smoking helps keep that feeling”; (g) “I enjoy parties more when I am smoking”; (h) “I feel more at ease with other people if I have a cigarette”; (i) “(Smoking helps me) in social situations”; (j) “I am afraid that I will be unable to function if I stop smoking”; (k) “I do better work when I am allowed to smoke”; and (l) “I feel like I am part of a group when I'm around smokers.” The scale was found to be reliable (Cronbach's alpha = 0.80). Perceived harms of cigarette smoking were based on four items from the *Smoking Consequences Questionnaire *[15]: (a) “Smoking is taking years off my life”; (b) “The more I smoke, the more I risk my health”; (c) “By smoking I risk heart disease and lung cancer”; and (d) “Smoking is hazardous to my health.” The scale was found to be reliable (Cronbach's alpha = 0.82). Response categories ranged from “strongly disagree” (coded as 1) to “strongly agree” (coded as 5), with higher scores indicating more health concerns.

 In the multivariate analyses, six demographic variables were included: gender (male versus female), age (continuous), race (white versus nonwhite), marital/relationship status (“involved” with someone versus not “involved”), educational attainment (college graduate versus less education), and monthly income (continuous).

### 2.3. Statistical Analysis

For the first part of the analysis (focusing on the onset of smoking behaviors), descriptive statistics are presented. In the second part of the analysis (examining the relationship between age of smoking onset and subsequent smoking behaviors), bivariate analyses were conducted. Correlation coefficients (Pearson's *r*) were computed to examine the relationship between initial smoking characteristics (age of smoking first cigarette, age of first purchasing cigarettes, and lapse time between first and subsequent cigarette smoking) and current smoking practices (number of cigarettes smoked, chain smoking, smoking while bed-ridden due to illness, smoking more than intended, making trips to procure cigarettes, needing cigarettes in order to function, perceived benefits of smoking, and perceived harms of smoking). In the final part of the analysis, multivariate analyses were undertaken to determine whether the age-of-onset measures were important contributors to the measures (a) number of cigarettes smoked and (b) smoking while sick in bed, when the effects of gender, age, race, marital/relationship status, income, and educational attainment were taken into account. These analyses were conducted via multiple regression, adding the age-of-onset measures alongside the demographic control variables. Throughout this paper, results are reported as statistically significant whenever *P* < .05.

## 3. Results

### 3.1. Sample Characteristics

 Slightly more than one-half (56.8%) of the study participants were male (*n* = 275). Participants ranged in age from 18 to 70, with a mean age of 36.4 (median = 34, SD = 12.3). Most of the respondents were either Caucasian (54.6%) or African American (39.0%) (*n* = 265 and 189, resp.). Most respondents (83.5%) were heterosexual (*n* = 405). Overall, this was a fairly well-educated research sample, with only 25.2% of the people having completed no more than a high school education (*n* = 122). Of those who had attended college (*n* = 363), nearly one-half (34.2% of the total sample) were college graduates or people with postgraduate education (*n* = 166). About one-half of the study participants were employed on a full-time basis at the time of their interview (55.1%); about one-third (32.9%) were employed on a part-time basis; and most of the remaining people (12.0%) were unemployed (*n* = 266, 159, and 58, resp.). On balance, this was a relatively low-income sample, with annual median income being approximately $21,500 (mean = $30,475, SD = $28,183, and range = $0 to $216,000). Compared to national data reported by the Centers for Disease Control and Prevention, the study sample included more women and was better educated. Otherwise, the sample reflected the characteristics of smokers nationally [16].

### 3.2. Age of Smoking Onset

The mean age at which people first reported smoking was 14.8 (median = 14, SD = 4.6). More than one-half (56.5%) of the study participants said that they smoked their first cigarette between the ages of 12 and 16, and the large majority (81.4%) reported having tried cigarettes before they were of legal age to smoke.

 Study participants reported purchasing their first cigarette approximately two years after they smoked their first cigarette (mean  age = 17.0, median = 16, SD = 3.9). More than one-half of the study participants (59.6%) said that they bought a cigarette for the first time between the ages of 15 and 18. Overall, slightly more than three-fifths of the study participants (61.9%) indicated that they were underage when they bought their first cigarette.

 After trying their first cigarette, approximately one-half of the study participants (53.2%) reported having smoked their next cigarette within one week. These persons were relatively evenly divided amongst those who said that the interval between their first and second cigarettes was less than one day (15.5%), approximately one day (16.8%), and more than one day but less than one week (20.9%). Another 18.0% of the study participants reported smoking their second cigarette within one month of their first one. Comparatively few people reported a first-to-second cigarette interval of one to three months (6.8%), three months to one year (6.8%), or a year or more (15.1%).

### 3.3. Initial Smoking Experiences

 Approximately one-half (51.6%) of the study participants said that they received their first cigarette from a friend. Another one-quarter (25.8%) said that they secretly took or stole their first cigarette from someone (e.g., a parent, another relative, or a friend). Much less commonly reported for the first cigarette has having purchased it on one's own (5.6%) or having gotten it from a sibling (4.5%), a relative other than a sibling or a parent (3.9%), or a parent (2.1%). 

 When asked to think back about their first time smoking a cigarette, approximately one-half (50.7%) of the study participants said that the experience was more negative than they had expected. Nearly one-third (30.2%) said that the experience was about what they thought it would be like, and the remainder (19.1%) said that it was more positive than expected. The most common negative experience remembered about their first time smoking was becoming dizzy (66.9%). Many study participants (53.7%) recalled that they disliked the taste of their first cigarette, while almost as many (52.1%) mentioned that it made them cough extensively. Conversely, approximately three-quarters of the study participants (77.9%) said that their first cigarette made them feel calm and relaxed. 

 When asked about the reasons for continuing to smoke after their initial cigarette, dominant explanations were the study participants felt that it was “cool” to smoke (57.5%) and they wanted to be with friends of theirs, who happened to be smokers (49.9%). Approximately one-quarter of the study participants (27.4%) said that they continued to smoke because they liked what cigarettes/smoking did for them. Peer pressure, in the form of being offered a cigarette from a friend whose offer they felt they could not refuse, explained continued use for 16.3% of the study participants. A comparable proportion of the people interviewed (16.1%) said that they decided to continue smoking after their initial experience because they thought that their subsequent experiences with cigarettes would be better than their first. Citing a liking for the taste of cigarettes as the reason for continued use occurred among 12.6% of the study participants.

### 3.4. Initial Smoking Experiences and Subsequent Smoking Behaviors and Perceptions

Table 1 presents Pearson's *r* correlation coefficients for the relationships between the three main age-of-onset measures (age of first cigarette smoked, age of first cigarette purchased, and lapse time between first and next cigarettes) and current smoking behaviors and smoking perceptions (e.g., number of cigarettes smoked, chain smoking, needing a cigarette in order to function, and perceived harms/benefits of smoking). The younger people were when they first smoked a cigarette, the more cigarettes they currently smoked (*P* < .05). Similarly, the younger they were when they first purchased a cigarette, the more they currently smoked (*P* < .001). Moreover, the shorter the interval between people's first and second cigarette smoking experiences, the greater their current tobacco use was likely to be (*P* < .10).

 Likewise, as Table 1 shows, the younger people were the first time they smoked, the more likely they were to smoke during adulthood when they were so ill that they could not get out of bed (*P* < .001). Similarly, the younger they were when they first purchased a cigarette, the more likely they were to smoke as adults when they were bed-ridden due to illness (*P* < .001). The shorter the interval between people's first and second time ever using cigarettes, the more likely they were, as adults, to smoke when they were too sick to get out of bed (*P* < .001).

Similar results are shown for needing a cigarette in order to function properly. The younger study participants were when they first smoked a cigarette, the more often they currently reported needing a cigarette to function properly (*P* < .05). A younger age of first purchased cigarettes was associated with a greater current need to smoke a cigarette in order to function properly (*P* < .001). Additionally, the shorter the period between the person's first and second time smoking cigarettes, the more likely the person was to report needing to smoke in order to function properly (*P* < .05). 

In contrast, initial smoking experiences were not found to be related to the frequency with which people made special trips to purchase cigarettes, chain smoking, smoking more cigarettes than intended, or with perceived negative health consequences resulting from smoking. As Table 1 shows, however, in terms of the perceived benefits of cigarette smoking, a younger age of cigarette smoking initiation and a younger age of first cigarette purchase were associated with perceiving more benefits from smoking in adulthood (*P* < .05 and *P* < .001, resp.). Additionally, the longer the interval between the person's first-ever and second-ever cigarettes, the more benefits of smoking the person perceived himself/herself to derive (*P* < .05). 

Table 2 presents the results of the multivariate analyses, which were undertaken to determine whether the age of onset measures that were statistically significant (as shown in Table 1) were robust enough to remain statistically significant when the effects of key demographic variables such as gender, age, race, marital/relationship status, educational attainment, and income were taken into account. Standardized regression coefficients (i.e., beta values) are provided so that relative effects sizes can be compared. In each instance, the initial smoking experiences measures (age of first cigarette smoked, age of first purchasing a cigarette, and time lapse between first and second cigarettes smoked) were found to be significant even when the demographic control variables were included in the analyses. The results for two of the main outcome measures are presented in Table 2; dependent variables not included in this table (e.g., chain smoking, perceived benefits resulting from continuing to smoke, smoking more than intended, etc.) were omitted in the interest of conserving space, but comparable findings were obtained for those measures as well. 

In this table, the dependent variables presented are the number of cigarettes smoked and smoking when one is so ill that one is bed-ridden. Both the age of first smoking and the age of first purchasing a cigarette were found to be statistically significant predictors despite the inclusion of two highly significant demographic control variables, namely, being Caucasian and being older, as well as the inclusion of gender, educational attainment, income, and marital status. The *R*-squared data indicate that more variance was accounted for with regard to the number of cigarettes smoked per week than smoking when ill and bed-ridden.

## 4. Discussion

Our findings show that the current smokers in our study typically smoked their first cigarette during adolescence, specifically in mid-adolescence. The mean age at which the study participants smoked their first cigarette was just shy of 15 years of age, with approximately one-half of all persons having initiated smoking behaviors between the ages of 12 and 16 years. This is a few years younger than the 17-18 years old age range reported by Fernander and colleagues [7] and Johnston and colleagues [1], but it is relatively close to the mean age of onset reported by Zabor and colleagues [17]. It is quite possible that people whose cigarette smoking ended with or shortly after initial experimentation, or people whose early-life smoking experiences did not turn into years-long smoking “habits,” began smoking at later ages than those who took part in the present study. The present study, in contrast, was characterized by persistent smokers who, on average, began their smoking careers at a young age. 

 Our findings pertaining to the interval between first tobacco use and subsequent smoking behaviors showed that, ordinarily, only a short period of time elapsed between smoking initiation or experimentation and the continuation of tobacco use practices. Nearly three-quarters (71.2%) of the people in this study reported having “progressed” from their first tobacco use incident to their second one in less than one month's time. This brief interval will make it very difficult for smoking prevention and intervention programs to have an impact, because they will have very little time to identify youths who are using cigarettes for the first time and then do something to intervene in their behaviors so as to prevent subsequent use. Parental vigilance and involvement in their children's (especially their teenage children's) lives, and acute awareness on the part of teachers and other school officials who have daily contact with youths, are likely to be the principal avenues by which early intervention can occur once smoking behaviors have been initiated. Previously published studies support this contention [18–22]. Educating parents and teachers about how to identify the signs that a young person is experimenting with tobacco and then providing them with strategies that they can use to broach the subject of smoking in an effective way with the youth(s) in question are essential if initial experiences with tobacco are to be prevented from turning into longer-term smoking behaviors. Supporting this type of approach, some studies have shown school-based smoking prevention/education/intervention programs to be effective at reducing tobacco use rates among children and adolescents [18, 23, 24].

 The present study also revealed that the initial smoking experiences of about one-half of the study participants were more negative than they expected. Consistent with other published reports [17, 25], most of the people taking part in this study said that their first time smoking caused them to feel dizzy, made them cough, and/or left them with a bad taste. Despite these negative experiences and sensations, they chose to smoke again anyway. Other researchers have found, as the present study did, that even negative initial experiences with smoking are related to subsequent smoking behaviors later in life [26]. Also of relevance here is the fact that many studies have found that large proportions of the people who eventually went on to become regular smokers experienced a variety of negative effects from their initial tobacco use, such as nausea, dizziness, and coughing (among others) [17, 26, 27]. More research is needed to understand the myriad factors that lead people whose initial smoking experiences are negative to continue to experiment with smoking and subsequently to become regular smokers. Peer pressure-related explanations alone do not explain this occurrence, because only about one-sixth of the study participants, thinking in retrospect, said that this was an important reason why they continued to smoke after their initial experiences using tobacco. The study findings show that peer influences or peer pressure to smoke was less powerful than what has been claimed in other researches, a finding that has been reported by others as well [28, 29]. Indeed, it may be that it is not so much peer pressure* per se *that leads young people to experiment with smoking as it is peer norms that are tolerant of smoking that eases adolescents and young adults into the process of normalizing their opinions regarding smoking practices, which in turn leads some of them to be less resistant to experiment with tobacco. Media messages that normalize or glamorize smoking may contribute to this process as well [28, 30, 31]. Likewise, parents who serve as in advertent role models for smoking behaviors or who provide weak or mixed messages with regard to adolescent smoking practices also play a role in fostering smoking-positive norms or belief structures for some youths, thereby increasing their odds of experimenting with tobacco [32–34].

 In our study, there were two primary reasons cited by study participants as to why they elected to smoke again after their initial experiences with cigarettes: perceiving smoking to be “cool” and wanting to spend time with friends who were smokers. These reasons are consistent with those reported by other researchers (e.g., [25, 35, 36]), who have mentioned such factors as enjoying “the buzz” created by smoking, smoking to cope with stress, considering smokers to look “cool” or to appear to be grown-up, enjoying the taste of cigarettes, feeling more “perked up” or alert after smoking, and enjoying the social/friendship aspects of smoking behaviors (among others) as being the main reasons cited by people in their studies for their initial use of cigarettes. Relating to the present study, youth-focused antismoking campaigns need to work to counteract messages about the “coolness” of smoking, and they need to provide realistic but scare-tactic-free messages about the “down side” to smoking. This latter point is particularly important because research has shown that health promotion efforts to reduce smoking among young people are less effective if they are too harsh with regard to their efforts to induce fear and/or disgust in their target audience [37]. That research demonstrated that the inclusion of some fear components or some disgust-inducing messages can be effective, but that using too much of this type of content is counterproductive. Developing fun, engaging, eye-catching, attention-keeping multimedia campaigns (e.g., online informational websites, and video games) that address smoking in a way that is age-appropriate and engaging for youths are likely to be effective ways of helping to curtail youth smoking. An example of one such program that has been shown to be effective [38] is the Adolescent Smoking Cessation Escaping Nicotine and Tobacco (ASCENT) Program, originally created by researchers at Danya International. Another example of a promising multifaceted, multimedia approach to preventing smoking among youths has been named ASPIRE (A Smoking Prevention Interactive Experience). It, too, has been shown to be effective [39]. Results from a mobile phone-based multimedia program to foster smoking cessation among youths have also demonstrated efficacy [40]. More innovative programs like these, which utilize multimedia platforms and take into account the need to be creative in the various ways they try to engage young persons in the smoking prevention/cessation process, are needed.

 Finally, we wish to discuss our findings pertaining to the age of onset of smoking behaviors and subsequent tobacco use practices. The strong tendency in this study was for earlier onset of smoking to be related to worse tobacco-related outcomes in adulthood. This is consistent with findings reported in the substance abuse literature generally (cited earlier) and in the tobacco literature specifically [9, 10, 12]). This finding highlights the importance of heading off early experimentation with cigarettes and to find ways to delay such experimentation to the greatest extent possible. Researchers and smoking prevention experts need to learn more about the factors that place people at risk for early-onset smoking. This is a topic about which little has been written (exceptions include the work of [9, 41]) and it is an area that would be fruitful for future researchers to explore. By learning more about who it is who is likely to experiment with smoking at an earlier age, we can increase the odds of reaching at-risk individuals and delaying (if not altogether preventing) their experimentation with cigarettes. Our findings suggest that, the longer this process can be delayed, the greater the likelihood is that better longer-term outcomes can be achieved.

 Before concluding, we would like to acknowledge four potential limitations of this research. First, the data collected as part of this study of adult persistent smokers were all based on uncorroborated self-reports. Therefore, the extent to which respondents underreported or overreported their involvement in various smoking-related behaviors is unknown. In all likelihood, the self-reported data can be trusted, as numerous authors have noted that persons in their smoking studies have provided reasonably accurate information about their tobacco-using behaviors [42–44].

 A second possible limitation pertains to recall bias. Respondents were asked to report about their beliefs, attitudes, and behaviors during the past 30 days or the past year, depending upon the measure in question. These time frames were chosen specifically: (1) to incorporate a large enough amount of time in the risk behavior questions' time frames so as to facilitate meaningful variability from person to person, and (2) to minimize recall bias. The exact extent to which recall bias affected the data cannot be assessed although other researchers collecting various types of smoking-related data have reported that recall bias is sufficiently minimal that its impact upon study findings is likely to be small [45]. This includes recall of earlier-life experiences pertaining to smoking onset [45].

 On this same subject, recall bias may also affect the data in terms of the amount of time that elapsed between people's initial smoking behaviors and the present. For example, on average, when people were responding to questions about their first time smoking, they were reporting on events that occurred slightly more than 20 years ago (mean = 21.6, SD = 12.6, and median = 20.2). As another example, when providing information about the first time that they purchased a cigarette, respondents were reporting on events that took place slightly less than 20 years ago (mean = 19.4, SD = 12.4, median = 17.5). Although initial usage of cigarettes is the kind of behavior that people oftentimes are able to remember fairly clearly [46], there is no way for us to know or assess specifically how accurate their recall is of these earlier-life events. Thus, the extent to which this aspect of recall bias affects the data used in this study is unknown. Other authors have addressed this issue in their own studies of smoking behaviors, however, and they have indicated that recall bias regarding earlier-life smoking behaviors appears to be minimal [45, 47].

 A third possible limitation of these data comes from the sampling strategy used. All interviews were conducted in the Atlanta, Georgia metropolitan area. There may very well be local or regional influences or subcultural differences between these persons and those residing elsewhere that could affect the generalizability of the data.

 A fourth possible limitation of these data comes in the form of potential cohort effects and the influence that this potential source of bias could have on the findings. Specifically of concern here is the influence that changing social attitudes towards smoking, along with the concomitant changes in social policies regarding smokers and smoking in public places, might have on the younger smokers (who grew up and began smoking in a relatively antitobacco culture) versus the older smokers (many of whom grew up and began smoking in a relatively prosmoking culture). To examine this possible source of bias in the data, we conducted additional ad hocanalyses of our data, dividing the sample into two groups: those aged 18–39 and those aged 40 and older. The multivariate analyses shown in Table 2 were then undertaken separately for the two age cohorts, to determine whether or not there was evidence of this type of bias in our data. Although of course the specific coefficients obtained in the multivariate analyses did change when we examined the data for cohort effects, the actual, substantive findings pertaining to the first smoking and first cigarette purchase experiences did not change. Thus, we did not find statistical evidence of cohort effects influencing our findings, which lends credibility to the data and the findings as they currently are presented in the paper and helps to quell concerns about the impact of cohort effects on this study's main findings.

## Figures and Tables

**Table 1:** Age of onset of tobacco use and subsequent smoking behaviors.

	Age of first cigarette use	Age of first cigarette purchase	Interval between 1st and 2nd cigarette
Number of cigarettes smoked per week	.14**	.19***	.08^†^
Smoking when so ill that the person is unable to get out of bed	.16***	.21***	.16***
Needing a cigarette in order to function	.11*	.16***	.09*
Making special trips to purchase cigarettes	.03	.07	.06
Chain smoking	.03	.06	.01
Smoking more cigarettes than intended	.03	.03	.04
Perceived benefits derived from continuing to smoke	.10*	.16***	.09*
Perceived harms resulting from continuing to smoke	.05	.02	.06

^†^ *P* < .10, *P < .05, **P < .01, and ***P < .001.

**Table 2:** Multivariate analysis for selected age-of-onset measures and adult smoking behaviors.

	No. of cigarettes smoked per week	No. of cigarettes smoked per week	Smoking when bed-ridden due to illness	Smoking when bed-ridden due to illness
Age of smoking first cigarette	−.12**	—	−.17***	—
Age of purchasing first cigarette	—	−.19***	—	−.22***
Gender (male)	.03	.03	−.06	−.07
Race (Caucasian)	.26***	.26***	.05	.05
Educational attainment (college graduate)	−.07	−.07	−.04	−.04
Income	.05	.05	.01	.02
Marital status (“involved”)	−.04	−.04	.02	.02
Age	.21***	.22***	.06	.08
*R*-squared	**.112 **	**.131 **	**.037 **	**.056 **

**P < .01, and ***P < .001.
